# Relationship between drug targets and drug-signature networks: a network-based genome-wide landscape

**DOI:** 10.1186/s12920-023-01444-8

**Published:** 2023-01-30

**Authors:** Chae Won Lee, Sung Min Kim, Soonok Sa, Myunghee Hong, Sang-Min Nam, Hyun Wook Han

**Affiliations:** 1 Department of Biomedical Informatics, CHA University School of Medicine, CHA University, Seongnam, 13488 South Korea; 2 Institute of Basic Medical Sciences, School of Medicine, CHA University, Seongnam, 13488 South Korea; 3 Department of Ophthalmology, CHA Bundang Medical Center, CHA University, Seongnam, South Korea

**Keywords:** Bioinformatics, Drug-gene network, Network analysis

## Abstract

Drugs produce pharmaceutical and adverse effects that arise from the complex relationship between drug targets and signatures; by considering such relationships, we can begin to understand the cellular mechanisms of drugs. In this study, we selected 463 genes from the DSigDB database corresponding to targets and signatures for 382 FDA-approved drugs with both protein binding information for a drug-target score (*KDTN*, i.e., the degree to which the protein encoded by the gene binds to a number of drugs) and microarray signature information for a drug-sensitive score (*KDSN*, i.e., the degree to which gene expression is stimulated by the drug). Accordingly, we constructed two drug–gene bipartite network models, a drug-target network and drug-signature network, which were merged into a multidimensional model. Analysis revealed that the *KDTN* and *KDSN* were in mutually exclusive and reciprocal relationships in terms of their biological network structure and gene function. A symmetric balance between the *KDTN* and *KDSN* of genes facilitates the possibility of therapeutic drug effects in whole genome. These results provide new insights into the relationship between drugs and genes, specifically drug targets and drug signatures.

**Supplementary Information:**

The online version contains supplementary material available at 10.1186/s12920-023-01444-8.

## Background

Drugs produce pharmaceutical and adverse effects according to the complex relationship between drug targets and drug signatures [1]. As gene analysis has become more prevalent, studies on the association between genes and drugs have also become more widespread in the field of drug research. As examples, Nagaraj et al*.* used a computational drug-repositioning approach to rapidly identify potent drug candidates for epithelial ovarian cancer treatment [2], Kim et al*.* assessed reversal gene expression profiles for gastric cancer using computational drug repositioning [3], and *Grenie and Hu* investigated drugs for inflammatory bowel disease using genetic information and computational methods [4]. In such studies, genes are divided into two categories: drug-target genes and drug-signature genes. Drug-target genes (also known as “druggable genes”) code for proteins that physically bind with the drug compound [5, 6], whereas drug-signature genes (also known as “drug-sensitive” genes) are differentially expressed due to drug–protein binding following drug treatment [1]. Studying the interactions between drug targets and drug signatures is important for drug discovery, drug repositioning, and identifying inference from potential adverse drug reactions [7, 8].

Ideally, the study of drug-related genes in both categories should extend to all human genes without any limitations. However, conventional studies have been limited to specific genes and specific drugs related to only a few diseases, and they have focused on only drug targets or drug signatures. Because such studies have investigated specific genes and drugs, the characteristics of all human genes in living cells are not typically taken into account. Additionally, for these reasons, conventional drug development studies may show effects on the phenotype of interest, and the presence of treated subjects is typically perceived as a cause of bias in genome-wide association studies [9]. Therefore, in the present study, we focused on the relationship between drug targets and signatures based on their characteristics. Our aim was to identify the genetic landscape via a multidimensional network using genome-wide drug–gene binding data and gene expression data.

In general, drugs affect the activity of proteins that correspond to target genes. During drug treatment, biological networks are disturbed and the expression of many other genes is significantly changed by unexpected responses to the drug. Thus, the pharmaceutical and adverse effects of the drug occur through complex relationships among drug targets and drug signatures [1]. Changes in gene expression by drug treatment can imply a therapeutic effect at the cellular level. From another perspective, drug treatment can have a perturbative effect in cells via gene networks [10]. Indeed, changes in gene expression in cells to maintain homeostasis arise due to perturbation [11].

To elucidate genome-wide inter-relationships between drug-target genes and signatures, we selected genes corresponding to targets and signatures for drugs that have both protein binding information for drug-target score (*KDTN*) and microarray signature information for drug-sensitive score (*KDSN*). *KDTN* represents the degree to which the protein corresponding to the gene binds to a large number of drugs, whereas *KDSN* represents the degree of the gene expression response following stimulation by drugs. Overall, we explored the network-based genome-wide landscape by comparing the cellular and functional characteristics of drug targets and drug signatures using the two variables *KDTN* and *KDSN*.

## Results

### Gene set analysis

#### Distribution of KDTN and KDSN in the DTSG set

**Fig. 1:**
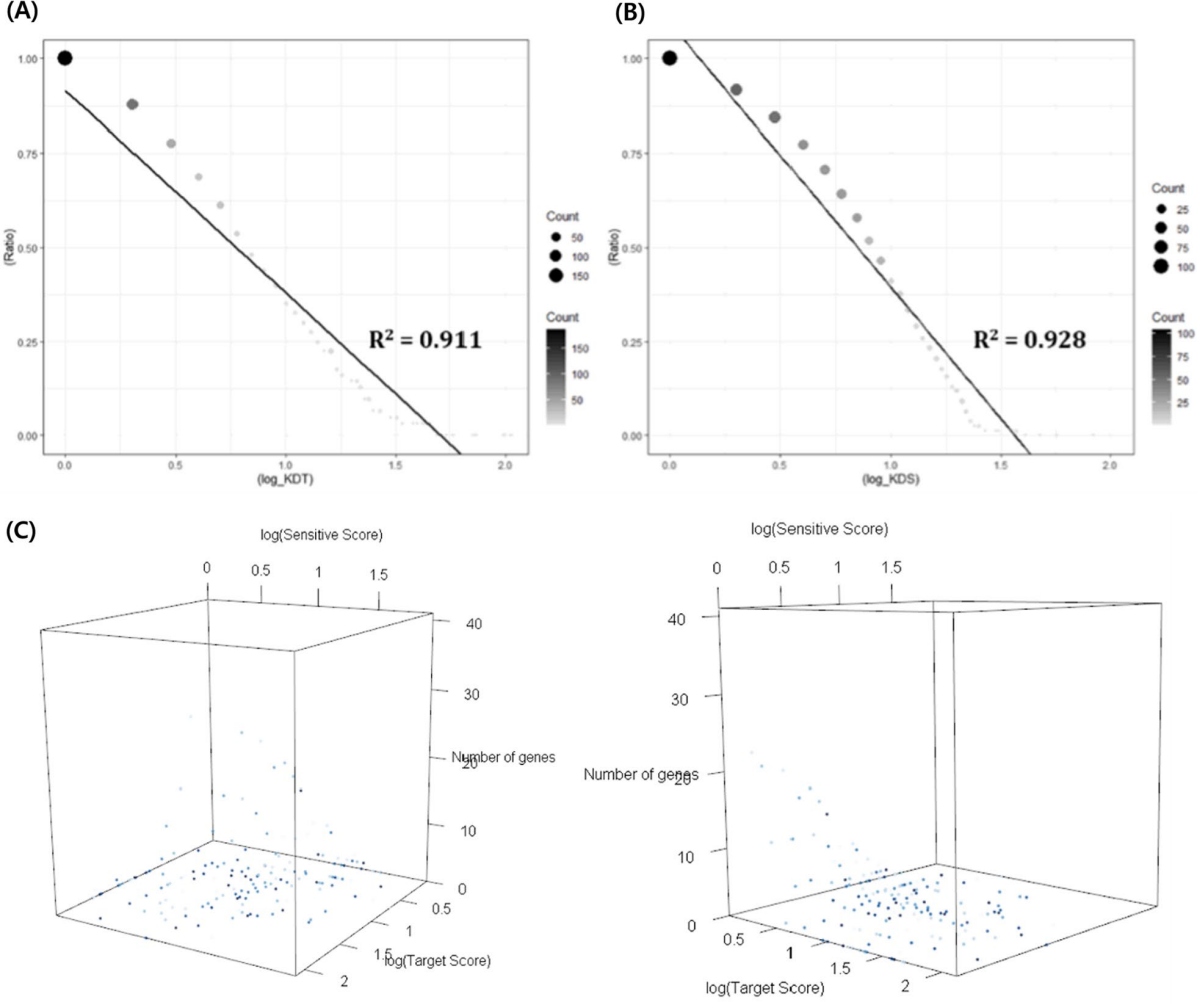
Degree distribution of the DTSG set. **A** Reverse-cumulative distribution of KDT within the DTSG set. **B** Reverse-cumulative distribution of KDS within the DTSG set. **C** Reciprocal relationships of the multidimensional network shown through three-dimensional plots

First, the distributions of *KDT* and *KDS* for the DTSG set were analyzed. Each distribution and the three-dimensional distribution for DTSG set were identified to elucidate the relationships between *KDT* and *KDS*. The three-dimensional plots demonstrate that the two distinct networks are reciprocally intertwined to constitute a curved surface (Fig. 1). As shown in Fig. 1, *KDT* and *KDS* had high dimensional scale-free and power-law distributions. It means genes highly connected in the drug-target network are least likely to be hubs in the drug-sensitive network, and vice versa [12].

#### Construction of the drug–gene network

**Fig. 2:**
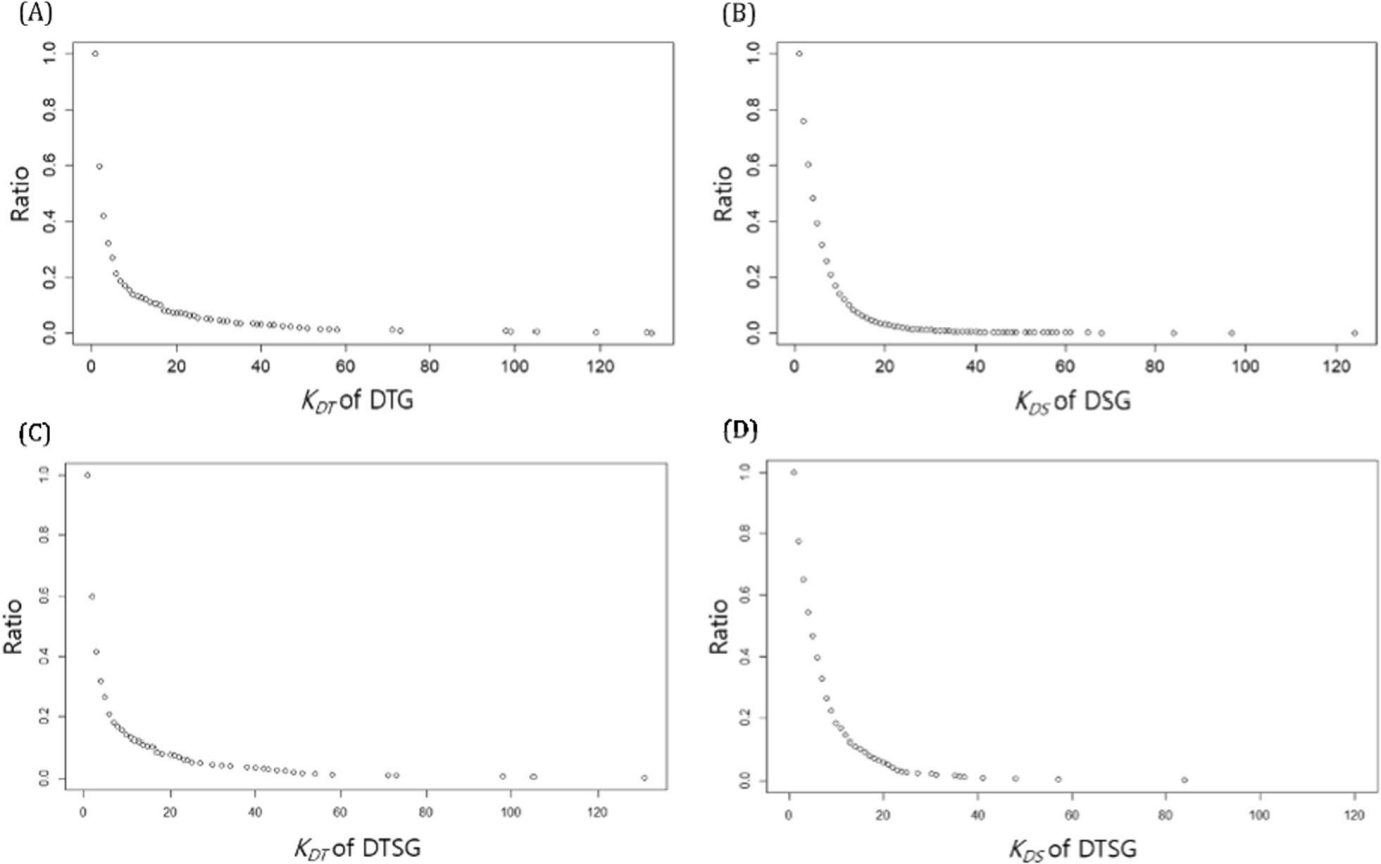
Reverse-cumulative distribution of *KDT* for D1 and the DSTG set; reverse-cumulative distribution of *KDS* for D3 and the DTSG set. **A** Reverse-cumulative distribution of *KDT* for 720 genes from the D1 target genes. **B** Reverse-cumulative distribution of *KDSN* for 9,233 genes from the D3 sensitive genes. **C** Reverse-cumulative distribution of *KDTN* for 463 genes from the DTSG set. **C** Reverse-cumulative distribution of *KDSN* for 463 genes from the DTSG set

The distributions of *KDT* and *KDS* for the DTSG set were compared with the D1 and D3 sets to determine whether the DTSG set had representativeness for the D1 and D3 set and to show the tendency of the DTSG set before a drug–gene network was constructed. Results showed that the distributions of *KDT* and *KDS* for the DTSG set presented representativeness for D1 and D3 (Fig. 2). Once representativeness was confirmed, an integrative drug–gene interaction network for the DTSG set and the drugs was visualized. However, visualizing all the target genes and sensitive genes made it difficult to intuitively observe the characteristics of the network. Therefore, only the target genes and sensitive genes for about 5% of the 371 drugs included in the DTSG set were analyzed by random selection, and we also offer whole drug-gene network of DTSG set as Additional file 2: Fig. S2.

**Fig. 3:**
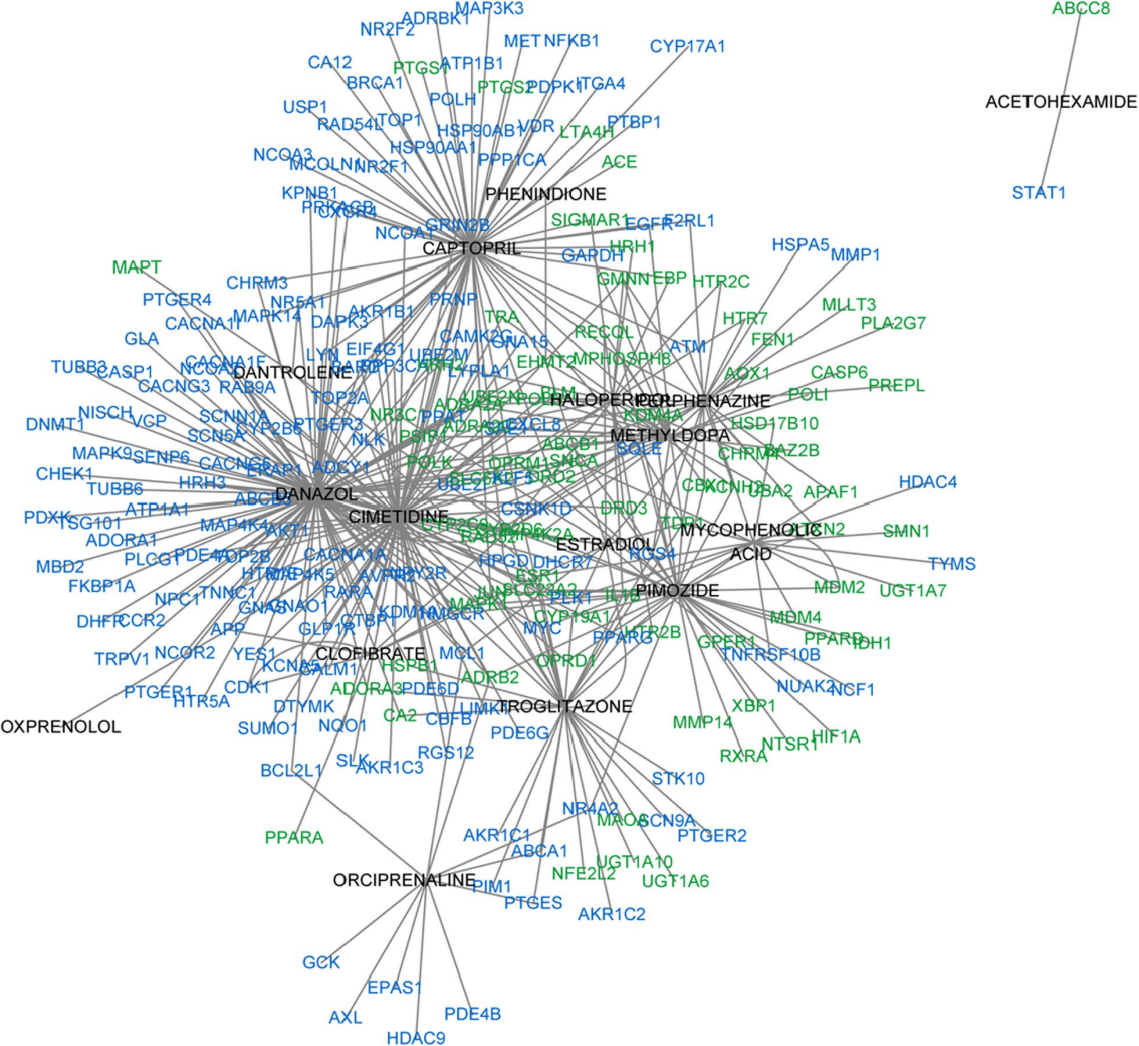
The drug–gene subnetwork of the DTSG set. The subnetwork of the drug–gene multidimensional network included about 5% of the drugs in the DTSG set. (black: drugs, blue: sensitive genes, green: target genes, purple: PTGER2)

The drug-target subnetwork and the drug-signature subnetwork were merged as a drug–gene subnetwork containing 253 nodes (16 drug and 237 genes) and 435 edges. As shown in Fig. 3 (in which the black-colored nodes represent drugs, the blue-colored nodes represent sensitive genes, and the green-colored nodes represent target genes), the relationships between the target genes and sensitive genes were exclusive and independent (Fig. 3). The whole drug-gene network described as Additional file 2: Fig. S1B.

### Enrichment analysis

GO/KEGG pathway [13] analysis for the *DSG, DTSG,* and *DTG* sets*.* Through GO analysis, the cellular components, biological processes, and molecular functions associated with each gene set were investigated. In the DTG set, 257 genes were associated with 13 cellular components, 28 biological processes, 20 molecular functions, and 11 KEGG pathways (FDR-adjusted *p*-value < 0.05). In the DSG set, 8,770 genes were associated with 41 cellular components, 63 biological processes, 21 molecular functions, and 8 KEGG pathways (FDR-adjusted *p*-value < 0.05). In the DTSG set, 463 genes were associated with 24 cellular components, 95 biological processes, 35 molecular functions, and 28 KEGG pathways (FDR-adjusted *p*-value < 0.05).

GO analysis revealed that most proteins synthesized by drug-sensitive genes were located in inner cellular zones such as the nuclear chromosome, nuclear pore, and nucleosome rather than in outer cellar zones such as the cell wall. The proteins synthesized by drug-sensitive genes were shown to be involved in gene transcription, gene expression regulation, and DNA replication, and to function in DNA, RNA, and protein binding. In contrast, GO analysis showed that most proteins synthesized by drug-target genes were located in outer cellular zones and played roles, for example, in receptor complexes, voltage-gated channel complexes, synapses, and cell junctions. Most proteins synthesized by drug-target genes were found to be involved in the catabolic process of cGMP and cAMP and in transmission and transport processes; they played roles in ion channels and enzyme activity.

**Fig. 4:**
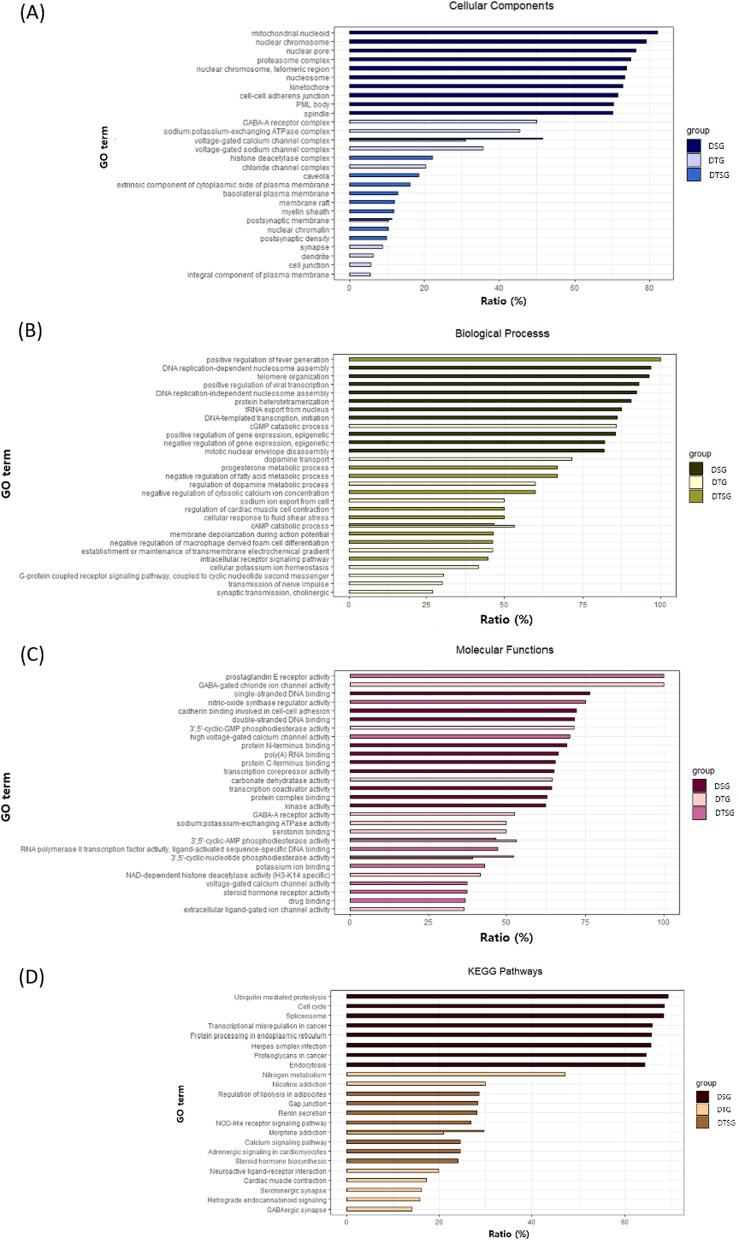
Gene Ontology analysis of each gene set. Top 10 GO terms for **A** cellular component, **B** biological process, and **C** molecular function from each gene set. **D** Top 8 KEGG pathway terms from each gene set. Through KEGG pathway analysis (figure), it was revealed that drug-sensitive genes were involved in central dogma-related pathways such as the spliceosome, transcriptional regulation, and protein-processing progress. However, drug-target genes were involved in neural signaling pathways such as addiction to nitrogen, nicotine, and morphine, serotonergic synapses, and retrograde endocannabinoid signaling

As shown in Fig. 4, only 6 terms (GO and KEGG) from 114 terms associated with the three gene sets (DTG, DSG, and DTSG) overlapped. Two cellular component terms overlapped in the DTG and DTSG sets: postsynaptic membrane and voltage-gated calcium channel complex. Of the molecular function terms, 3′,5′-cyclic-nucleotide phosphodiesterase activity and 3′,5′-cyclic-AMP phosphodiesterase activity overlapped in the DTG and DTSG sets. From the biological processes terms, only one term overlapped between the DTG and DTSG sets: cAMP catabolic process. Similarly, one KEGG pathway term, morphine addiction pathway, overlapped in the DTSG and DTG sets. These results suggest that drug-target genes and drug-sensitive genes are exclusive and independent in terms of their cellular locations, genetic functions, processes, and pathways.

### Transcription factor (TF) analysis

**Fig. 5:**
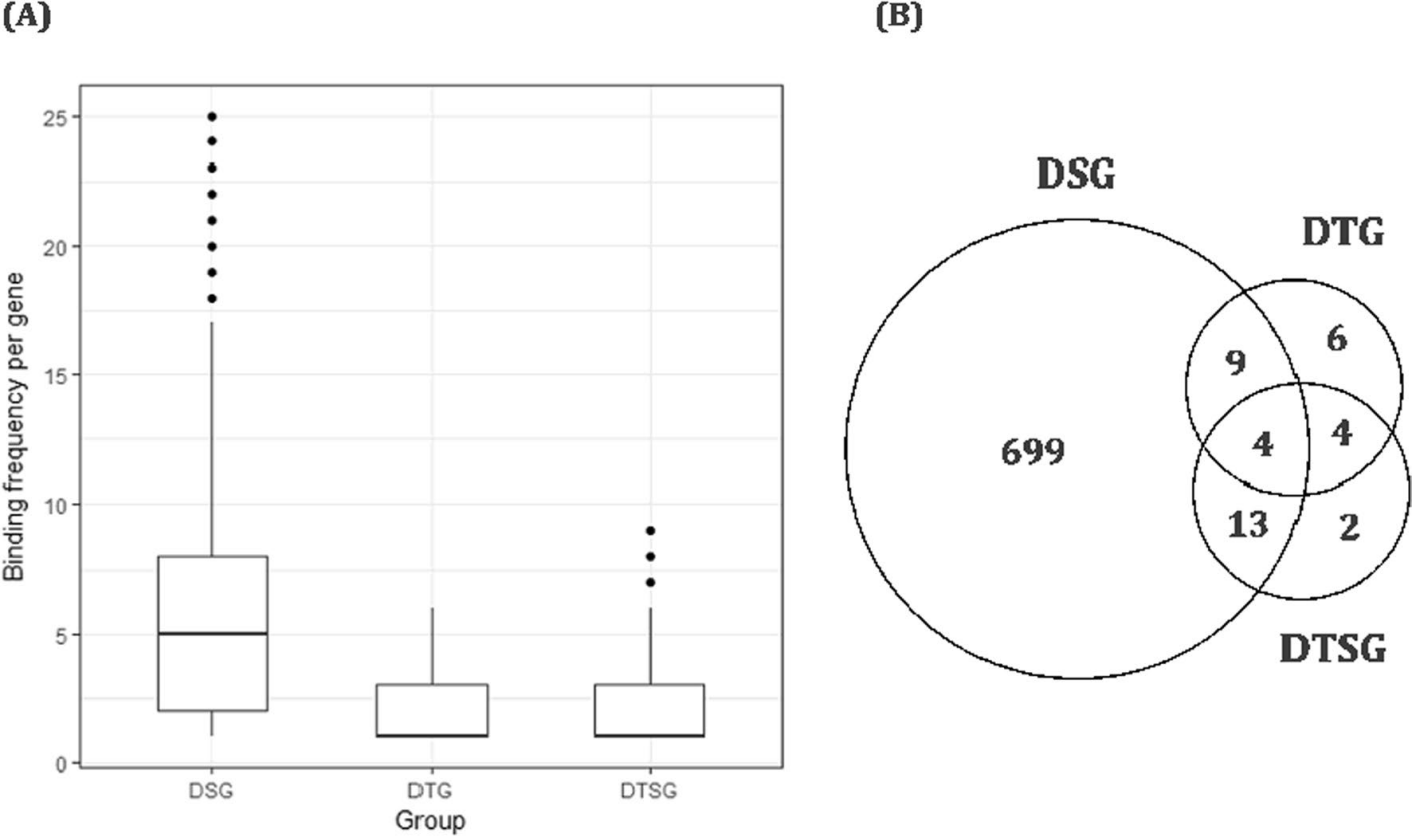
(**A**) Binding frequency of transcription factors per gene involved in the DSG, DTG, and DTSG sets. **B** The number of transcription factors derived from the DSG, DTG, and DTSG sets

In gene set analysis, it is important not only to characterize the gene set but also to identify the number and type of TFs as this can help to improve understanding of gene regulatory networks. Thus, we examined whether there were differences in the number of TFs involved in each gene set (Fig. 5b). We used X2Kweb [14] as a TF analysis tool to examine the binding frequency and types of TFs for each gene set. Results showed that TFs bound on DNA strands on average six times per gene in the DSG set, which was three-fold greater than the TF binding in the DTG and DTSG sets (both two times per gene on average, Fig. 5a). In total, 737 TFs were associated with the three gene sets. Of these, 30 TFs overlapped between two or more gene sets as shown in Fig. 5b. Therefore, the TFs involved in each gene set differed. Of the 30 overlapping TFs, 9 were derived from essential genes in humans (< 10% of all human genes are considered essential) [15].

### Core gene analysis of DTN and DSN

**Fig. 6:**
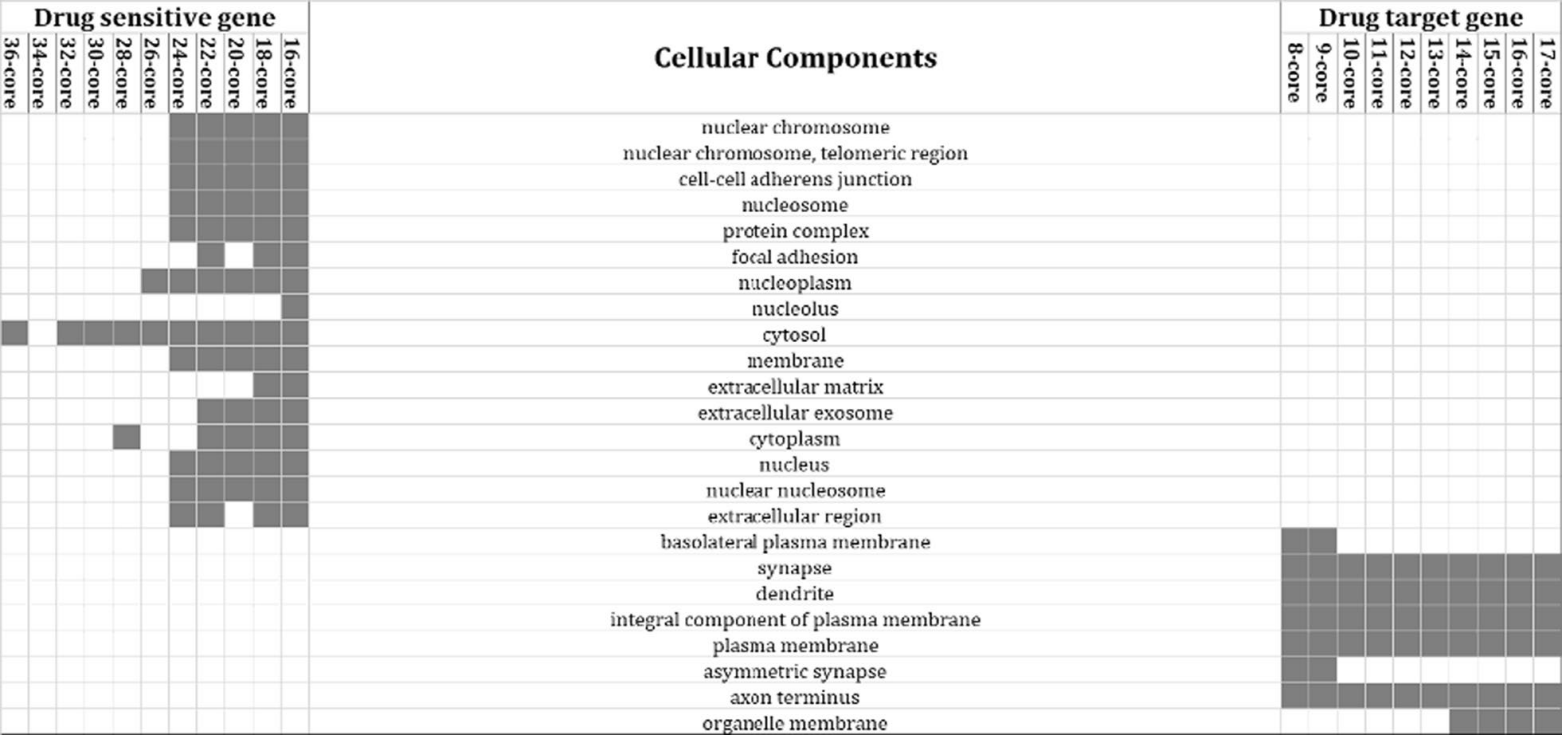
Cellular component analysis of the core genes from each gene set

Characterization of the core genes in *KDT* and *KDS* for the DTG and DSG sets was examined by applying a peeling algorithm. Each network that included > 50 genes was analyzed according to *m*-core. *m*-core of network is defined as a maximal connected subgraph of network in which all vertices have a degree of at least *m* [12]. As a result, m-coreDSN had 1 to 36 core gene groups whereas m-coreDTN had 1–17 core gene groups. Figure 6 indicates the gene ontological characterization in each network according to *m*-core. In cellular component analysis, the core genes of each DSN and DTN showed exclusive distributions. Proteins synthesized by core genes of the DSN were located in the cytosol, cytoplasm, nuclear chromosome, and nucleosome. Conversely, proteins synthesized by the core genes of the DTN were located in the synapses, dendrites, plasma membrane, and axon terminus. In molecular function analysis, the core genes of each DTN and DSN were also exclusively distributed. Proteins synthesized by the core genes of the DSN functioned during cell–cell adhesion and in protein heterodimerization activity by binding proteins and cadherin. The proteins synthesized by the core genes of the DTN functioned in ion binding, hormone binding, chemical receptor activity, and enzyme activity functions (Additional file 1: Fig. S1A). In biological processes analysis, the core genes of the DSN and DTN networks also showed exclusive distributions. Proteins synthesized by the core genes of the DSN were involved in the PERK-mediated unfolded protein response, response to hypoxia, positive regulation of angiogenesis, and regulation of cell death. In contrast, proteins synthesized by the core genes of the DTN were involved in the response to drugs, dopamine transport, receptor signaling pathways, and monoterpenoid metabolic processes (Additional file 1: Fig. S1B).

In summary, characterizations of genes in the DSG, DTG, and DTSG sets in terms of GO and KEGG pathways could clearly be distinguished. In addition, the numbers and types of TFs differed among the DSG and DTG sets with different binding frequencies of the TFs on DNA strands. Finally, *m*-core analysis of the core genes in each *DSN* and *DTN* exhibited reciprocal balanced characteristics.

## Discussion

Here, we investigated the relationships between two major gene groups, i.e., target and signature genes, according to various perspectives related to drug discovery and development. Our purpose was not only to investigate the relationship between DTG and DSG sets but also to demonstrate the genome-wide landscape of drug–gene multidimensional networks based on relationships between DTNs and DSNs. We classified genes into three sets based on their operational roles such as their physical binding and/or response to drugs. First, we constructed DTNs and DSNs from experimental data such as drug-target and drug-signature data. Subnetworks were constructed using experimental data from drug targets and drug signatures. In a multidimensional network model constructed by merging each subnetwork, the *KDTN* and *KDSN* of the DTG, DSG, and DTSG sets were calculated; thus, the mean network interaction degree of each subnetwork was calculated. The three gene sets, namely DTG, DSG, and DTSG, were compared in four ways: (1) the distribution between *KDTN* and *KDSN*, (2) GO and KEGG pathway analysis, (3) the number and type of TFs, and (4) GO analysis with *m*-coreDSN and *m-*coreDTN according to *KDSN* and *KDTN*.

Sorting gene processes using D1 and D3 showed that drug targets are not usually affected by the drug as reported in a previous conventional study [16]. Therefore, only 463 genes intersected between 720 DTG and 9,233 DSG sets. The degree distributions of *KDTN*, *KDSN*, and subnetwork visualization showed that the relationship between the DTG and DSG sets was exclusive. Each degree of *KDTN* and *KDSN* showed a power-law distribution and their relationship was reciprocal. This is indirect evidence that the response of cells to drugs is structured and organized systematically [17, 18].

The relationships of GO terms (cellular component, molecular functions, and biological process) and KEGG pathways between the DTG, DSG, and DTSG sets was also reciprocal. This shows that the DTG and DSG sets have distinct functional differences in cells. Thus, studies to investigate target genes, not signature genes, should be conducted according to aspects of GO and KEGG pathways, as shown in the current study.

TF analysis of each gene set showed that the average binding frequency of TFs involved in the DSG set was six times per gene. The TFs involved in each gene set were also exclusive and different. Thus, genes acting as drug signatures seem to be regulated with binding frequencies three-fold greater than those of genes acting as drug targets. Consequently, genes regulated with 1–3 TFs would be good candidates for drug-target genes.

GO analysis using the *m*-core of each network showed that the functional and spatial characteristics of the target gene core and the signature gene core differ. Mutually exclusive characteristics were also exhibited.

In conclusion, the expression of target genes was barely affected by drug treatments. Therefore, the pharmaceutical effect of drugs was due to the DSGs for which expression levels were significantly changed by drug treatment rather than the direct action of DTGs. These complex drug–gene relationships can produce drug side effects as well as therapeutic effects. This study provides a potential new approach to discovering drugs. However, further studies are needed to identify the therapeutic effects and adverse drug reactions associated with the relationship between the DTN and DSN.

## Methods

### Data

The drug and genome database DSigDB (http://dsigdb.tanlab.org/DSigDBv1.0/) [19] is an open-source database that currently includes 22,527 gene sets and consists of 17,389 unique compounds covering 19,531 genes. Gene sets provide seamless integration by which to link gene expression with drugs/compounds. DSigDB organizes drugs and small molecule-related gene sets into four domains based on data for drug-induced quantitative inhibition and/or changes in gene expression. The data from DSigDB contains four domains (D1–D4) that collect drug and genome data for four purposes, as shown in Table 1. The D1 and D3 domain datasets were used to construct each drug-target network (DTN) and drug-sensitive network (DSN) in this study.

**Table 1:** DB table from DSigDB

Domain	Purpose
D1	A total of 1,202 Food and Drug Administration (FDA)-approved drugs including 1,288 target genes. Here drug-target gene refers to the gene-coded proteins that physically bind and interact with drug compounds
D2	In total, 1,220 kinase inhibitors (1,065 unique kinase inhibitors) covering 407 kinases that frequently mutate in various cancers
D3	Gene expression profiles obtained by induction with compounds. In total, 7,064 gene expression profiles were collected from three cancer cell lines perturbed by 1,309 compounds from CMap (build 02) [20]. Compounds that were profiled by multiple cell lines were unified and genes with > twofold change relative to the control (either up- or downregulation) were considered as gene sets
D4	In total, 10,830 and 5,163 gene sets were compiled from the Therapeutics Targets Database [21] and the Comparative Toxicogenomics Database [22], respectively, which were extracted from literature using a mixture of manual curation and text mining approaches

### Construction of the network

FDA-approved drugs for which both protein binding data and microarray experiment data were available were used from the DSigDB database. Following these principles, the D1 and D3 datasets were used and matched to their drug and gene ID terms.

**Fig. 7:**
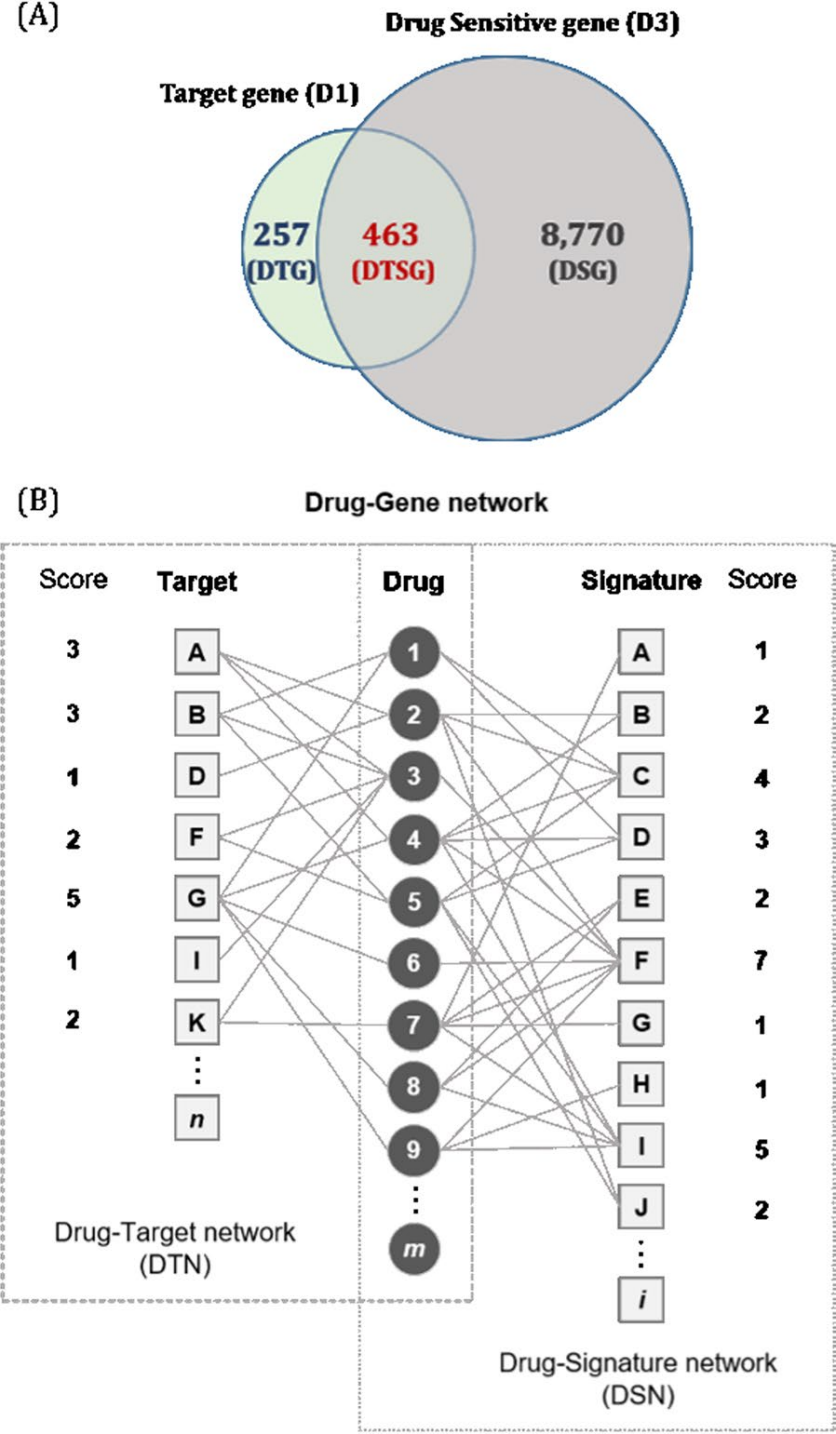
Construction of the drug–gene network. **A** Venn diagram of gene groups and the number of genes in each gene set for 382 drugs (DTGs: drug-target genes; DTSGs: drug-target and sensitive genes; DSGs: drug-sensitive genes). **B** Process of the drug–gene network construction

In the process of matching terms from each domain, the Pubchem compound term (https://pubchem.ncbi.nlm.nih.gov/) [23] and Entrez term (https://www.ncbi.nlm.nih.gov/Web/Search/entrezfs.html) [24] were used as the drug and gene ID. Consequently, 382 drug compounds and 9,490 genes intersecting with D1 and D3 were extracted (Fig. 7A). Using these data, the DTN and DSN were constructed from calculated gene scores, i.e., *KDTN* and *KDSN,* based on the relationship between drugs and genes as follows:

$$T_{j} = (KDTN) = \sum\limits_{j} {t_{ij} }$$

$$\left(\begin{array}{ll}{t}_{ij} &=1 ({\mathrm{Drug\, i\, binds\, gene\, j}})\\ &= 0 {\text{(else)}}\end{array}\right)$$

$$S_{i} = (KDSN) = \sum\limits_{j} {s_{ij}},$$

$$\left(\begin{array}{ll}{s}_{ij} &= 1({\text{adjust}}\, p{\text{-value}} < 0.05\, {\text{and}}\, {\text{FC}}>2\, {\text{or}}\, {\text{FC}} < 0.5)\\ &= 0 {\text{(else)}}\end{array}\right)$$

where the range of *KDTN* and *KDSN* was from 1 to 186 and from 1 to 104, respectively, and the number of genes with *KDTN* ≥ 1 and *KDSN* ≥ 1 was 720 and 9,233, respectively. Among these genes, 463 simultaneously had both *KDTN* ≥ 1 and *KDSN* ≥ 1. From the relationship between the *KDTN* and *KDSN* of genes, the genes were divided into three groups as shown in Fig. 1A: the DTG set (drug-target genes: 257 genes), DSG set (drug-sensitive genes: 8,770 genes), and the DTSG set (drug-target and -sensitive genes: 463 genes).

Figure 7B shows the process by which a drug–gene network was constructed. Two kinds of bipartite network (DTN and DSN) were constructed, which were then merged into a multidimensional network. For example, Gene A binds to three drugs among nine drugs in the DTN (drug 2, 3, and 4) but its expression was changed by only one drug in the DSN (drug 7). Using such analyses, the target score and drug-sensitive score of each gene from the binary network model were calculated.

### Analysis

Gene enrichment analysis and network analysis were conducted in three groups of genes (DTG, DTSG, and DSG) using Gene Ontology (GO) and Kyoto Encyclopedia of Genes and Genomes (KEGG) pathway analyses via DAVID (https://david.ncifcrf.gov/) [25]. The top 10 GO terms and top 8 KEGG pathway terms for each gene group, i.e., those terms that were most enriched, were determined and are shown in Fig. 4. The distributions among *KDTN* of D1, *KDSN* of D3, and *KDTN* and *KDSN* of DTSG were compared using the ggplot package in R. We then constructed a drug-target bipartite network for the drugs and genes involved in the DTSG set using Cytoscape. For network visualization, 19 drugs and 170 genes were used; these 19 drugs represented about 5% of the 371 drugs included in the DTSG set. Furthermore, network analysis was conducted using the concept of *m*-core decomposition to analyze the central function according to *KDTN* and *KDSN.* A “peeling algorithm” aims to characterize a network hub and elucidate the relationships between nodes based on network connectivity [12]. In a multidimensional network, the nodes represent the drugs and genes of the DTG and DSG sets, respectively, and the edges represent the relationships between drug-target genes or drug signatures. In the present study, we applied a peeling algorithm represented by *m*-core [12]. Specifically, *m*-coreDSN and *m*-coreDTN are defined as the maximal connected subgraph of the DSN and DTN, respectively, in which all genes have a degree of *KDSN* and *KDTN* greater than the *m* value.

## Supplementary Information

**Additional file 1. Supplementary figure 1.** Molecular function and biological process analysis of the core genes from each gene set.

**Additional file 2. Supplementary figure 2.** Whole drug-gene network of DTSG set. (Red : Drug, Blue : DTG, Green : DSG)

## Data Availability

The datasets used and/or analysed during the current study are available from the corresponding author on reasonable request.

## Abbreviations

KDSN: Degree of Drug-Sensitivity Network
KDTN: Degree of Drug-Target Network
D1: Domain 1 (DSigDB)
D2: Domain 2 (DSigDB)
D3: Domain 3 (DSigDB)
D4: Domain 4 (DSigDB)
