# Supplementary material for: Relationship between drug targets and drug-signature networks: a network-based genome-wide landscape
Source: BMC Med Genomics. 2023 Jan 30;16:17. doi: 10.1186/s12920-023-01444-8 (PMC9885570; doi:10.1186/s12920-023-01444-8)
Supplement: Supplementary file 1 — Additional file 1. Supplementary figure 1. Molecular function and biological process analysis of the core genes from each gene set. [file 12920_2023_1444_MOESM1_ESM.docx]

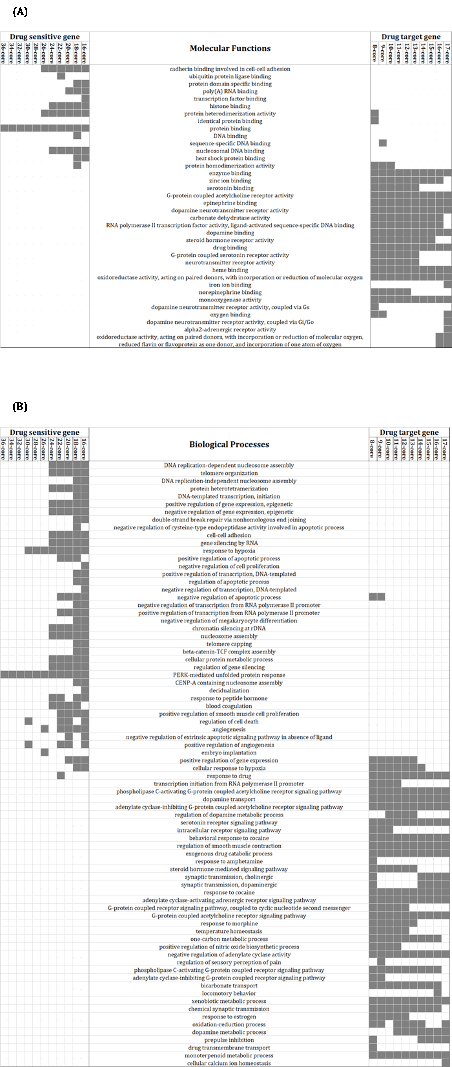


Supplementary figure 1. Molecular function and biological process analysis of the core genes from each gene set

(A) Molecular function analysis of the core genes from each gene set (B) Biological process analysis of the core genes from each gene set
