# Supplementary figures and images for: Relationship between drug targets and drug-signature networks: a network-based genome-wide landscape
Source: BMC Med Genomics. 2023 Jan 30;16:17. doi: 10.1186/s12920-023-01444-8 (PMC9885570; doi:10.1186/s12920-023-01444-8)

*
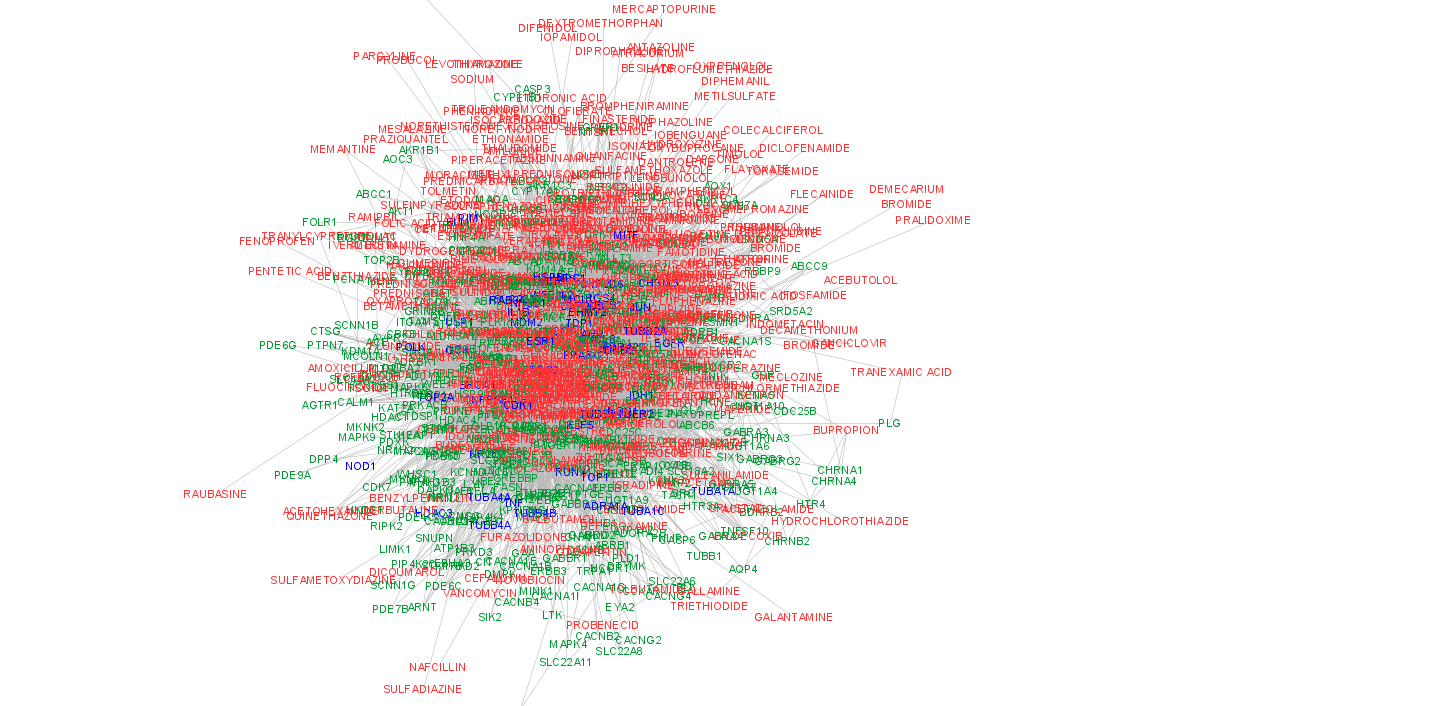
*

Supplementary figure 2. Whole drug-gene network of DTSG set. (Red : Drug, Blue : DTG, Green : DSG)

Supplement: Supplementary file 2 — Additional file 2. Supplementary figure 2. Whole drug-gene network of DTSG set. (Red : Drug, Blue : DTG, Green : DSG) [file 12920_2023_1444_MOESM2_ESM.docx]
